# Elementary Treatise on Diagnosis, Prognosis, and Therapeutical Indications; a Course of Clinical Medicine

**Published:** 1827-01

**Authors:** 


*Traité Elémentaire de Diagnostic, de Pronostic, d'Indications Therapeutiques; ou Cours de Médecine Clinique.* Par M.L.Rostan, Médecin de l'Hospice de la Vieillesse, (femmes) ci-devant Sal Petrière, Professeur de Médecine Clinique, &c. Tome premier.

The work which we now introduce to the notice of our readers embraces one of the most important of medical inquiries—*the*

*principles of clinical medicine;*—and it involves the discussion of many topics of the very highest interest, especially to the young medical student. We shall, in this review, follow our author nearly in the course which he has chosen, though we greatly fear we shall not always come to the same conclusions.

The work is divided into two parts:—1. Preliminary observations; 2. The diagnosis. The latter part is subdivided into two chapters—the first relating to symptoms, the second, to symptoms as signs of disease—a sub-division, which is surely an over-refinement, and leads to much repetition; symptoms are always signs, and it is entirely owing to the defective state of our knowledge that we do not always certainly know what is signified by them.

### PRELIMINARY OBSERVATIONS.

M. Rostan begins his useful work by the statement and elucidation of what appears to us to be certain self-evident, and we had almost said, undisputed propositions. At least we regard them so to be on this side the Channel. It is plain, however, by the tone of our author's publication, that that is by no means the case in the meridian of Paris. These propositions are, 1. That there can only exist, in the animal frame, *organs* and *functions;* 2. That the latter are *effects* of the former; and, 3. That according as, and *only* as, the organs are healthy or unhealthy, so are the functions. When the organs are healthy, the functions are invariably so too, and together they constitute healthy anatomy and physiology; when the organs are unhealthy, the functions participate with them, and these together constitute morbid anatomy and physiology.

Surely all this is undeniable. What is intended, then, by the division of morbid affections into functional or organic?—or into disorders and diseases? And, is this division founded in nature and useful in practice? There is certainly a vast difference between dyspepsia, gastritis, and scirrhus of the stomach: in the first, no change can generally be detected in the organs on inspection after death—in the second, certain changes in colour, if not in texture, are usually obvious enough—and, in the last, there is altogether a change in substance; in the first, even, it has been observed that the disorder has sometimes led to organic disease, although it must be admitted that it has more frequently subsisted for many years, and even for a long life, without any tendency, even, to superinduce disorganization. It is, therefore, quite obvious that there is a vast difference in these cases; it is equally obvious that it must be of vast importance to ascertain this difference in actual practice; and yet it must, we think, be admitted to be a misuse of terms to denominate the case of dyspepsia, functional, as distinguished from that of the other two morbid affections, as organic; for in both there is an affection of the organ as the foundation and substratum of that of the function. But, we would ask, is there the same or any objection to the terms disorder and disease, as applied, the former to cases in which no change can be observed on dissection, the latter to such cases as consist in an obvious change of texture? We think not. For these terms do, each, express changes of the organ, although changes which are very different, in point both of fact and importance. We would call that, disorder or derangement, which cannot be seen on examination of the organ after death, and that, disease, which is obvious under the scalpel of the anatomist; and it would, of course, be an important object in the *history* carefully to trace the transitions from one state to the other. We do not know whether these plain, but surely practical remarks, may satisfy the philosophical turn of mind of M. Rostan, who has taken great, and, we think, unnecessary pains, to explode all idea of diseases of function independent of diseases of the organ; such ideas, we think, never prevailed amongst us; and for the distinction of disorder and of disease, it is of too useful a character, as well as too substantially correct, to be abandoned. We must, therefore, continue to use the term disorder, for the periphrase—"organic affection not detectible on examination," and the term disease for "organic affection obvious on inspection"—considering both terms, however, as expressing changes of the *organ*, and coincident changes of its *function*.

We have, perhaps, bestowed too much pains upon this discussion, but it is plain that M. Rostan regards the point as one of mighty importance; nay, he goes so far as to assert, that the future progress of medical science depends upon our having just views of this question; but the kernel of all his remarks is this, that the more we search for disease, in cases in which it has been thought there was none to be detected, the more likely we shall be to discover it,—and that we should always *suspect* it, and so *look for* it, and especially in those cases which have hitherto been denominated *nervous*, and, as it appears, in Paris, *vital*. M. Rostan, in his ardour to explain that there cannot exist either function or disordered function independently of organ, proposes (p. 7) to say that there is no such thing as respiration or digestion, but rather "respiring organs," "digesting organs;" and of course, and for the same reason, there can be no disorder, but disordered organs, no disease, no death, but diseased and dying or dead organs, &c.! all which is perfectly undeniable; and yet we greatly fear we shall never be able to induce our brethren here to speak with such becoming propriety.

It is quite obvious, from the style of M. Rostan's performance, that it is controversial. The first part is, for this very reason, rather tedious to us who are strangers to the author's antagonists; we shall, however, state briefly the five "*propositions*" which it is intended to establish. The first is, that there are, in the animal economy, only organs, and organs in exercise—or functions; the second, that each organ may be affected primarily and independently; the third, that our fluids are also susceptible of primary morbid change; the fourth, that it is impossible that there should be only one kind of morbid affection; the fifth, that the strength varies in each individual, and, consequently, that the treatment must be very various, and sometimes contrary. These propositions require no proof amongst us, and it is surely very deplorable that they should amongst our enlightened neighbours; we had almost thought that M. Rostan was in joke, but he appears to be quite serious, and he is very animated in his observations on these different points.

M. Rostan proceeds to state that it is by *observation* alone that the truth of these facts is demonstrated, and that it is by means of the *senses* alone that we observe, and obtain all our information. These subjects have already been fully noticed by us in a former Number,* to which we refer our readers. We proceed, therefore, to the discussion of another question agitated by M. Rostan;—*what is the most important kind of knowledge for the physician?* Is it the knowledge of the causes of disease? of the intimate nature of disease? of the symptoms? No. None of these—but, according to M. Rostan, that of the morbid anatomy. Now, we have several times lately given our opinion on this subject; and we have surely never attempted to depreciate the true and just value of morbid anatomy; but we are far, very far, from thinking that an undivided, or even unequal, attention should be paid to this study. We would say, in a few words, that the symptoms and the morbid anatomy should *together* engage the chief attention of the physician;—for without the symptoms as the signs of the disease, of what utility would be the most intimate knowledge of diseased structure? and without an accurate knowledge of the morbid

* Vol. 2, No. for April, 1825, p. 415.

anatomy, how can the symptom ever be to the physician the *sign* of the disease? In this point of view, then, the knowledge of the symptoms and of the morbid anatomy, is of *equal* necessity and importance. But the symptoms teach us much more; they teach us, 1. The degree of strength of the patient, and, 2. the condition of the constitution—two points of indispensable importance to the treatment. For it is utterly impossible to prescribe for a disease merely, supposing it even most accurately known; we must first inquire most carefully into those other circumstances of the patient which we have just mentioned. It has been well remarked that all works on agriculture should be re-written, stating the nature of the *soil;* and that all works on physic should be re-written, noticing the state of the strength and of the constitution; and it may even be a question, whether we might not as safely be ignorant of the disease itself as of these other important points.

M. Rostan endeavours to prove that the study of morbid anatomy is the most useful, because the works on this subject are the most permanent in their value and fame, and instances the works of Bonnet and Morgagni, of Senac, Corvisart, Bayle, &c. But this is a very ambiguous mode of reasoning. Does M. Rostan mean by the word useful—useful in actual practice and to the patient, or to the reputation of a medical author? If the latter, we believe we should quite agree with him, and this for obvious reasons. First, because the observation and the description of the morbid anatomy are far easier than those of the objects of symptomatology; secondly, because the changes in morbid structure, are of a more solid and tangible, and lasting, and far less variable character, than those of the functions constituting the signs of disease; and it is, therefore, far easier to write accurately upon them. Yet the difficulty of a study by no means lessens its *utility;* and he who, out of the more transitory and chaotic mass, can collect substantial observations, has even the greater merit; and it may, with great truth, be asserted, that the work of Sydenham has had a more important and useful influence on the practice of physic than even that of Morgagni.

The study of the symptoms is essential, then, to make even our knowledge of the morbid anatomy available and useful—to indicate the degree of strength of the patient, and to ascertain other peculiarities of the individual constitution; it is further of *sole* efficacy in that great number of cases in which the anatomist has hitherto traced no organic changes. It is also a study of greater difficulty than that of morbid anatomy.

Our younger readers will not now, therefore, hesitate to determine which study should claim their most special regard; if their object be to become authors, and to acquire great fame merely, we think they had better, for these purposes, study the morbid anatomy;—but if they wish to become useful to their patients, they will do well to bestow their chief attention upon the symptoms—not an undivided or exclusive attention, however; but carefully adding to this study that, also, of the morbid anatomy.

The question we are now discussing—what is the most important study for the physician?—involves many other considerations, besides those which we have hitherto touched upon. There is another inquiry, distinct from that into the symptoms and the morbid anatomy, which has hitherto been too much neglected; it is that which relates to the *history*. This inquiry involves the consideration of the following questions:—1. Hereditary predisposition; 2. Previous diseases; 3. Preceding attacks of the same disease, or of disease of the same cavity or organ; 4. The course of the disease, especially the *first* or *earliest* effects of *insidious*, and the *premonitory* signs of *impending* diseases,—the *extension*, or the *transition*, of disease from one organ to another, or from one cavity to another,—the *superinduction* or the *supervention* of other diseases, or of diseases of other parts,—the *changes* in the character of the existing disease, &c.; 5. The effects of remedies; and, 6. Of external circumstances, as exertion, emotion, diet, &c. M. Bayle, of whose character M. Laennec has drawn so beautiful a portrait,* was so impressed with the importance of an accurate attention to these points, that he was in the habit of taking a faithful account of the disorder in every case, and of leaving it with the patient when his attendance was concluded, for the benefit of any future medical adviser. Certainly, if this plan were rightly pursued, there might be some foundation for that boasting which we sometimes meet with in the older medical attendants of a family, and which has gone by the phrase of "knowing the constitution"—a phrase, of the frequent abuse of which to sinister ends, we cannot here treat more particularly.

It is, then, from an accurate inquiry into the history, and from an accurate observation of the symptoms, that we are led to ascertain the disease, the course, and the actual state, of that disease, and the precise condition of the patient, that is, of the constitutional powers and actions; on this knowledge depends our treatment entirely; it involves that of the morbid

* Vol. ii. p. 109.

anatomy; but it applies, also, to innumerable cases in which no morbid changes have hitherto been detected on dissection.

Now, then, let us endeavour to appoint to the morbid anatomy its due place in a course of medical study. It is surely quite indispensable; it has thrown the greatest light upon medical science; it constitutes the most permanent and substantial part of medical writings. Morbid changes of structure should be suspected and looked for, in every case, and, when no such change can be discovered on dissection, it should be frankly confessed that they have eluded our modes and means of observation,—not that they do not exist. The investigation should be prosecuted with ardour, and opens the most certain way to fame. But let it be repeated, that not the most accurate and minute knowledge of morbid structure can avail the physician in curing or relieving the patient, unless he can ascertain it in the *living* person, as well as in the dead; so that its actual utility is just commensurate with our knowledge of the history and symptoms, or of the signs by which we are led to ascertain its existence.

It will be observed that, in the preceding observations, we have not said one word which should lead our readers to deny the utility of the study of morbid anatomy; our object has been to assign to it its due place in medical studies. M. Rostan has, however, collected some observations supposed to be made by such as feel disposed to depreciate morbid anatomy as a study, not in the comparison with other studies, but in itself. These objections are the following:—

1. "Many changes which did not exist during life, may supervene after death.

2. "Many others, on the contrary, which existed during life, may disappear—as the redness of erysipelas.

3. "It is a folly, as the ancients, and Celsus in particular, observe, to suppose that the appearances after death are the same as during life: and—

4. "The fluids may become morbidly affected, and the changes in them cannot be supposed to be detectible on dissection."

These are certainly important observations, and ever to be borne in mind in our investigations into the morbid anatomy, but are surely no objection to the study itself, nor do they in the least degree lessen its value. They lead us to make a remark, however, which is of the utmost importance; that it is absolutely necessary to be intimately acquainted with the *healthy* appearances of each of the organs constituting the animal frame, as examined under different circumstances, at different periods after dissolution, &c. before we can be prepared to ascertain the morbid anatomy. There are appearances which our neighbours have distinguished and denominated "cadaveriques;" the work of M. Laennec abounds in observations upon them; but he would do a most essential and lasting service to the science of medicine, who would treat the matter systematically and fully. If any of our readers be so minded, we would recommend to their perusal two excellent papers, one by Dr. Yelloley, the other by Dr. Davy, in the Medico-Chirurgical Transactions, and they will not find the following observations altogether useless.

1. How difficult is it to discern what is, and what is not morbid, in the degree of *fulness of the vessels?* We have only to open the head and examine the substance of the brain, or the chest, and inspect the state of the lungs,—or even to read many published accounts of such examinations, fully to comprehend the difficulty of this question. The most experienced eye alone can distinguish the apparently, from the really, morbid states of fulness, and we may even suspect the truth of all such conclusions as have been drawn from those appearances alone.

2. How important, yet how difficult, is the same question in regard to *colour*. The nature of this difficulty will be best apprehended on opening the abdomen, and examining the different hues observed in the course of the stomach and intestines. How often has inflammation and even gangrene, been pronounced to have existed, when there had been nothing less.

3. How difficult, too, is the question respecting the *size* of certain organs, as of the heart or of the liver. We must be well acquainted, indeed, with the natural appearances and differences, to be enabled to offer a correct opinion upon the question, whether either of these organs be larger or smaller than natural, when the alteration is only slight.

These examples will suffice to draw the attention of our readers to the inquiry proposed, and to explain our meaning. To the investigation of these and similar topics must be added that of those pointed out by M. Rostan, and then, and then only, shall we be prepared to ascertain and distinguish the really morbid changes which may be presented to us.

Having thus accurately distinguished between the healthy and diseased structures, there are other points in the farther inquiry into the diseased, of the greatest importance, but which we can only touch upon cursorily at this time. In the really morbid appearances observed, it is essential to ascertain at what period of the patient's life or sickness they took place. Whether they belong to previous disease or diseases,—to the earlier or later stages of the fatal disease,—whether they co-exist accidentally, or have occurred as causes and effects of each other,—and especially whether they have been induced in the last stage of all, or have been the effect of the state of sinking, &c. &c. The last point is of the utmost importance as well as novelty. It is noticed more than once in M. Laennec's immortal work, but altogether demands a renewed investigation.

The next subject brought under our review by M. Rostan, is that of the *mental qualifications* necessary to the study of physic. These he enumerates thus: first, *attention;* in the second place, *an ardent and pure love of truth;* in the third place, *a state of moderate scepticism,* equally avoiding prejudice on the one hand, and incredulity on the other.

The attention must not only be great, but it must be kept up, and that even after we have come to our conclusions respecting the nature of the case. For who is the physician who has never had occasion to correct and change his opinion during his continued attendance on a patient? and how shall he be apt to correct his opinions unless his attention be continually kept alive to fresh or varied appearances in the course of the disease? And then, admitting our first opinions to be just, how often does the disease itself change both its form and its seat, or become complicated by the superinduction or supervention of others; and how shall these changes be instantly detected except by a strict and sustained attention?

But if the error which results from a defective attention be serious, it is at least pardonable in comparison with that which is the effect of prejudice or of the want of a pure and simple love of truth. Here is scope for discipline of the heart as well as of the head. Our readers remember that when the celebrated Dr. Sangrado was told that all his patients died, and that he must, therefore, alter his system of bleeding and warm water, the doctor replied, "impossible, for I have written a book upon it." It is a sad snare to have written a book. It is rarely that men are, like Sydenham, above a prejudice or bias produced by their own writings. But we have already treated of this subject, and will dismiss it by referring to what we have already written.*

A doubting spirit, is also that which keeps the attention alive. He who knows little, doubts little, and remains perhaps in error; whilst he who has learnt to doubt, continues to observe, and corrects or confirms his own opinions by a well

* The Number for April, 1825, p. 419.

sustained attention and observation; his ultimate opinions being thus formed, are well formed, and stable in proportion.

The next subject, treated of by M. Rostan, are the *therapeutic indications*. Of these we need not treat at large. Our author's remarks contain little or no novelty and scarcely admit of animadversion. In stating the diagnosis to be the *basis* of the treatment, M. Rostan concurs with the author of the only treatise on this subject in the English language, and, we believe, with *every* intelligent practitioner on this island. The sources of the therapeutic indications are thus enumerated.

"1. The causes of the disease.
2. The nature of the disease.
3. The course and duration of the disease.
4. The strength of the patient.
5. The age of the patient.
6. The constitution of the patient.
7. The sex, and
8. The habits, and idiosyncrasies."

These subjects are treated of very cursorily, and are too important to be so treated of. The same may be said of a little section on the "means we possess to fulfil these indications."

We shall next, therefore, proceed to notice the second part of M. Rostan's work,—that to which the part already noticed is, indeed, merely introductory. But as the character of this second part is altogether more *practical* than the first, we shall reserve it for a second article in our next; especially as we wish to condense upon our pages, all the useful observations it contains. In this part of our review we have enlarged freely upon our author's observations; in the next, we purpose to adopt a different course:—viz. to compare M. Rostan's account of the symptomatology with that contained in the only work on this subject in our language;—we mean the Essay on the Symptoms and History of Diseases, by Dr. Marshall Hall—to transfer to our pages such observations as appear to form useful additions to those contained in Dr. Hall's work, and to intersperse such remarks as may tend, we think, to the advancement of this important department of medical science.

